# Validation of the English Version of the Scale for Psychosocial Factors in Food Allergy and the Relationship with Mental Health, Quality of Life, and Self-Efficacy

**DOI:** 10.1155/2016/4850940

**Published:** 2016-09-05

**Authors:** Rebecca C. Knibb, Aaron Cortes, Christopher Barnes, Carol Stalker

**Affiliations:** ^1^Psychology, School of Life and Health Sciences, Aston University, Birmingham, UK; ^2^Orthopaedic Department, Universidad de Chile Clinical Hospital, Santiago, Chile; ^3^Psychology, College of Life and Natural Sciences, University of Derby, Derby, UK

## Abstract

*Background*. The Scale for Psychosocial Factors in Food Allergy (SPS-FA) is based on the biopsychosocial model of health and was developed and validated in Chile to measure the interaction between psychological variables and allergy symptoms in the child. We sought to validate this scale in an English speaking population and explore its relationship with parental quality of life, self-efficacy, and mental health.* Methods*. Parents (*n* = 434) from the general population in the UK, who had a child with a clinical diagnosis of food allergy, completed the SPS-FA and validated scales on food allergy specific parental quality of life (QoL), parental self-efficacy, and general mental health.* Findings*. The SPS-FA had good internal consistency (alphas = .61–.86). Higher scores on the SPS-FA significantly correlated with poorer parental QoL, self-efficacy, and mental health. All predictors explained 57% of the variance in SPS-FA scores with QoL as the biggest predictor (*β* = .52).* Discussion*. The SPS-FA is a valid scale for use in the UK and provides a holistic view of the impact of food allergy on the family. In conjunction with health-related QoL measures, it can be used by health care practitioners to target care for patients and evaluate psychological interventions for improvement of food allergy management.

## 1. Introduction

Food allergy affects approximately 6–8% of children worldwide [1]. It is thought that prevalence rates might be rising, particularly in the UK which has one of the highest rates of food allergy in the world [2, 3]. Prescott et al. collected data from 89 countries, including already published data, and reported that for food allergy diagnosed by oral food challenge the UK had the highest prevalence for children over five years of age and the fourth highest prevalence for children under five after Australia, Norway, and China [4]. An allergic reaction to food involves the release of histamine which causes unpleasant symptoms such as a skin rash, swelling of the mouth or face, gastrointestinal symptoms such as vomiting, and in severe cases difficulty in breathing and anaphylaxis [1]. There is no cure for food allergy and management is through strict avoidance of the food. Studies over the last twenty years have shown that food allergy is related to poorer quality of life in both patients and carers [5, 6]. Social quality of life including family activities, holidays, and school trips and emotional and psychological quality of life are particularly affected areas [5, 6]. Food allergy has also been associated with high levels of stress and anxiety in parents, particularly mothers [7–12], and in some cases high levels of depression [12].

A number of food allergy specific scales have recently been developed to more accurately measure the impact food allergy has on the life of the patient and the carer. These include scales to measure quality of life of the patient [13–15], parental burden [16], and self-efficacy for food allergy management [17]. In a recent study, greater parental self-efficacy (confidence) in management of food allergy for their child was shown to relate to better parental quality of life [17]. Many of these scales have now been translated and validated in different languages, facilitating cross-cultural research, which shows how food allergy has an impact on families across different countries [18]. The Scale for Psychosocial Factors in Food Allergy (SPS-FA) was developed and validated in Chile [19] to measure the impact of caring for a child with food allergy that takes into account the interaction between psychological variables and allergy symptoms in the child. It is based on the biopsychosocial model of health [20] which recognises the bidirectional relationship between biological, psychological, and social factors related to a long-term condition such as allergy. The development of the SPS-FA was based on research showing that acute stress can increase an immune response triggering symptoms such as asthma [21–23] and chronic stress can trigger a Th2-type immune response, upregulating the immune system and increasing levels of inflammatory markers such as eosinophils, which can trigger symptoms association with allergic conditions such as atopic dermatitis [24, 25].

The SPS-FA aims to provide a more holistic view of the impact of food allergy in the parent/child dyad. It was demonstrated to have excellent reliability and was significantly associated with anxiety and depression in the parent. The aim of this study was to validate an English version of the SPS-FA and explore the relationship between scores on this scale and parental quality of life, self-efficacy for food allergy management, and mental health in parents caring for a child with food allergy.

## 2. Methods

### 2.1. Design

This was a cross-sectional study using validated psychometric scales to measure the psychosocial impact of food allergy (SPS-FA), food allergy specific parental quality of life (FAQL-PB), food allergy specific parental self-efficacy (FASE-P), and parental mental health (GHQ12). Ethical approval for this study was provided by the Psychology Research Ethics Committee at the University of Derby, UK (102-13-CB). All participants gave written informed consent to take part.

#### 2.1.1. Participants and Procedure

Participants were parents from the general population in the UK, who had at least one child with a clinical diagnosis of food allergy. They were recruited through the Anaphylaxis Campaign Charity's website and associated social media (e.g., Facebook and Twitter). Participants had to be parents of at least one child under the age of 18 years living in the family home who had a food allergy diagnosed by a clinician at an allergy clinic through clinical history and either skin prick tests, blood tests, or food challenges. All participants completed the questionnaires online using Survey Monkey®, an online survey platform which allows participants to complete questionnaires from their PC or tablet.

### 2.2. Scale Translation

The Scale for Psychosocial Factors in Food Allergy (SPS-FA) [18] was originally developed in Spanish and translated into English by the author of the original scale (AC) and checked by two native English speakers (RK and CB) to ensure the translation was grammatically and syntactically correct. A number of minor changes to wording were made and the scale was rechecked by the original author in order to ensure that the wording of the English version was a match for the original Spanish version. The SPS-FA is a 14-item scale which is completed by a parent to measure the psychosocial impact of food allergy in the child and caregiver dyad. The scale has three subscales: quality of life (*n* = 6 items), crisis (*n* = 5 items), and social impact (*n* = 3 items). The original version developed in Chile had an overall alpha of .87 with subscale alphas of .816 for quality of life, .805 for crisis, and .787 for social impact [19]. Items are scored on a 0–4 scale from not at all to very much. Scores are summed and divided by the number of items in each subscale to get a mean total for each subscale with a score range of 0–4. Higher scores indicate a greater psychosocial impact of food allergy.

### 2.3. Cross-Sectional Validation Measures

#### 2.3.1. Food Allergy Quality of Life-Parental Burden (FAQL-PB) Scale [16]

The FAQLQ-PB has 17 items and uses a 7-point Likert scale ranging from 1 (not troubled) to 7 (extremely troubled). Questions include issues concerning going on vacation, social activities, and worries and anxieties over the previous week. A higher score indicates greater parental burden. Internal validity has been reported as excellent in a US sample (Cronbach *α* = 0.95) [16] and in a UK sample (*α* > 0.85) [26].

#### 2.3.2. Food Allergy Self-Efficacy Scale for Parents (FASE-P) [17]

The FASE-P asks parents how confident they are in managing their child's food allergy. It has 21 items and uses a 100-point scale where parents rate themselves from 0 (cannot do at all) to 100 (highly certain can do). Questions cover five subscales: (i) managing social activities, (ii) precaution and prevention, (iii) allergic treatment, (iv) food allergen identification, and (v) seeking information about food allergy. A higher score indicates greater self-efficacy for food allergy management. The FASE-P scale has excellent internal consistency (Cronbach's *α* = 0.88) and good validity [17].

#### 2.3.3. Food Allergy Independent Measure (FAIM) [27]

The FAIM has 4 items which measure the severity of perceived risk of an accidental reaction to food and the risk of not being able to treat a reaction appropriately. It has been used as a means of measuring the impact of food allergy in the validation of other related questionnaires such as the Food Allergy Quality of Life Scales [13–15]. Items are answered on a 7-point Likert scale with a greater score indicating a higher level of perceived seriousness.

#### 2.3.4. General Health Questionnaire-12 (GHQ-12) [28]

The GHQ-12 is a 12-item scale of current mental health which asks individuals to state how they have felt over the last few weeks. It measures inability to carry out normal functions and also the appearance of new and distressing symptoms. It uses a 4-point Likert scale from not at all (scored 0) to much more than usual (scored 3). Scores are summed and have a range from 0 to 36. Scores over 11-12 indicate a risk of being diagnosed with a mental illness. The scale has excellent internal consistency (Cronbach's *α* = 0.77–0.93) and good validity [28].

### 2.4. Discriminative Validity

#### 2.4.1. Demographic and Food Allergy Questionnaire

A questionnaire to gather demographic information from the parent and food allergy information about their child was developed based on that used in previous published studies [29]. Information collected included the type of food allergy, symptoms, how the allergy was diagnosed, medication, history of anaphylaxis, and presence of other atopic conditions such as asthma, hay-fever, and eczema.

### 2.5. Statistical Analysis

Data analyses were conducted using SPSS (version 22, SPSS, Chicago, IL, USA). Missing data was treated pairwise, apart from missing data for psychometric scales which was treated listwise in order to ensure that missing items did not affect overall scale scores. Internal consistency of the SPS-FA was assessed using Cronbach's *α* coefficient and Guttman's split-half coefficient. Pearson's bivariate correlations were conducted between scale scores to assess construct validity. A priori hypotheses were set regarding reliability and validity following guidelines provided by Pesudovs et al. [30]. We expected Cronbach's alpha of >0.7 and <0.9 and moderate convergent validity correlations of >0.3 with subscales measuring similar aspects to the SPS-FA such as quality of life. Between-subjects *t*-tests and Pearson's correlations were performed to assess the discriminative validity of the SPS-FA by comparing demographic and food allergy characteristics. Pearson's correlations and multiple regression models were conducted to explore the relationship between the SPS-FA, quality of life, self-efficacy, and mental health. All tests were 2-tailed with alpha set at ≤0.05.

## 3. Results

Demographic and food allergy information for participants can be found in Table 1.

### 3.1. Reliability of the SPS-FA

The SPS-FA scale had excellent internal consistency with an overall Cronbach's *α* of 0.86. Split-half Cronbach's *α* was .82 for part 1 and .73 for part 2; the Guttman split-half coefficient was 0.81. Alphas for the subscales of the SPS-FA were moderate to excellent: quality of life = .82; crisis = .61; social impact = .80.

### 3.2. Cross-Sectional Construct Validity

Higher total scores on the SPS-FA representing a greater psychosocial impact of food allergy on the parent/child dyad significantly correlated with poorer parental quality of life (QoL) (*r* = .68), poorer self-efficacy (*r* = −.48), and poorer mental health in the parent (*r* = .46). It was also significantly correlated with greater expectation of a serious food allergic reaction in their child (*r* = .29) (Table 2). The subscales of the SPS-FA were also significantly correlated with QoL, self-efficacy, mental health, and expectation of outcome (Table 2).

### 3.3. Discriminative Validity

There was no significant difference in SPS-FA scores between parents of girls with food allergy compared to parents of boys. Greater psychosocial impact of food allergy was significantly correlated with younger age of the parent (*r* = −.18, *p* < 0.001) and younger age of the child (*r* = −.14, *p* < 0.01). SPS-FA scores were also correlated with the number of food allergies the child had (*r* = .25, *p* < 0.001) showing that the more allergies the child had the greater the psychosocial impact of the food allergy (Table 2).

Parents whose child had suffered from anaphylaxis reported a greater psychosocial impact of food allergy compared to parents whose child had not (Table 3); similarly parents whose child had been admitted to hospital because of food allergy reported a higher psychosocial impact compared to parents whose child had not (Table 3). For children with asthma as well as food allergy, parents reported significantly greater psychosocial impact of food allergy compared to children with no concomitant asthma. This was also the case for eczema (Table 3), but not for hay-fever.

There were no significant differences in SPS-FA scores for parents of children who had allergies to peanuts or other nuts compared to those who did not. Parents of children who had allergy to cow's milk or to egg reported significantly greater psychosocial impact of food allergy compared to parents whose children did not have reactions to milk or egg (Table 3). There was also a greater psychosocial impact for each of the subscales of the SPS-FA for parents of children who had anaphylaxis, had been hospitalised, had asthma, had eczema, and had cow's milk or egg allergy (all *p* < 0.05, data not shown).

### 3.4. Relationship between SPS-FA, Mental Health, Self-Efficacy, and Quality of Life

The relationship between demographic and food allergy characteristics, the psychosocial impact of food allergy, mental health, self-efficacy, and quality of life was explored using a hierarchical multiple regression model with the psychosocial impact of food allergy (SPS-FA scores) as the outcome variable. In the first step demographic and food allergy variables that were significantly associated with SPS-FA scores were entered in order to control for these variables. The model was significant (*F*(9,334) = 5.97, *p* < 0.001) and explained 12% of the variance in SPS-FA scores; the number of food allergies, asthma, and attendance at hospital were significant predictors (Table 4).

In the second step, quality of life, self-efficacy, mental health, and expectation of outcome scores were entered. The model was significant (*F*(13,330) = 33.91, *p* < 0.001) and explained 56% of the variance in SPS-FA scores (Table 4). Only the number of food allergies retained its significance from the first step; quality of life, mental health, and self-efficacy all significantly predicted the psychosocial impact of food allergy in the second step. Quality of life had the biggest standardised beta, demonstrating the strong positive relationship between psychosocial impact of food allergy and food allergy specific quality of life.

## 4. Discussion

This study aimed to assess the reliability and validity of the English version of the SPS-FA and explore its relationship to quality of life, self-efficacy, and mental health of the parent. We found the SPS-FA to be a valid and reliable scale to use in a UK population. Reliability analysis showed the scale to have excellent internal consistency with an overall alpha the same as that of the original scale [19]. The subdomains quality of life and social impact also had good-to-excellent internal consistency; however the crisis subscale had a slightly lower alpha to that of the original scale. The SPS-FA demonstrated good validity with significant correlations in line with expectations. There were moderate-to-strong correlations with mental health, replicating results reported in the development of the original scale where there were significant correlations with anxiety and depression [19]. A greater psychosocial impact of food allergy on the parent/child dyad was strongly related to poorer food allergy specific quality of life. This is not surprising given that the SPS-FA has a quality of life subscale, but the correlation was less than 0.7 demonstrating that the SPS-FA measures additional aspects of the impact of food allergy. The SPS-FA also correlated with self-efficacy, signifying that greater self-efficacy for management of food allergy was related to less psychosocial impact of food allergy. This is encouraging and suggests that improving self-management might reduce the impact of food allergy on the parent/child dyad.

The SPS-FA showed good discriminative validity, demonstrating the scale's sensitivity to the severity of food allergy. Greater psychosocial impact related to a greater number of food allergies, history of anaphylaxis, hospitalisation, and concomitant asthma and eczema. These variables have been reported in other studies to be associated with QoL [13, 15, 31] and add to the literature highlighting the importance of these factors in explaining the impact food allergy might have on the family. Age of parent and child was also related to SPS-FA scores with younger parents and parents of younger children reporting a greater psychosocial impact of food allergy. This has also been found in studies looking at the impact of food allergy on the quality of life of parents and children [32]. Knibb et al. [17] found that parental self-efficacy for food allergy management is higher in older parents and older children with food allergy and it may be that a greater confidence and ability to manage allergy as the child gets older reduce the psychosocial impact of the allergy. Age was not predictive of SPS-FA scores; therefore the relationship between age and the psychosocial impact of food allergy warrants further investigation to assess cause and effect. This study also found that the psychosocial impact of food allergy was greater for those children with milk and egg allergy. This has been reported in relation to quality of life and self-efficacy [17] and highlights the difficulties parents face when trying to manage these types of allergies.

Regression analysis showed that only a relatively small proportion of scores on the SPS-FA could be explained by food allergy characteristics alone and only the number of food allergies retained its significance when quality of life, self-efficacy, mental health, and expectation of outcome scores were entered. Food allergy specific quality of life had the strongest relationship with SPS-FA scores and suggests that reducing the psychosocial impact of food allergy might improve food allergy specific quality of life. As this is a cross-sectional study we cannot make any predictions about causation and a longitudinal study assessing the psychosocial impact of food allergy from the point of diagnosis and over time would help to interpret these findings more clearly and provide information on the longitudinal validity of the scale. We were not able to conduct a test-retest of the SPS-FA and so stability over time is a factor that still needs to be ascertained for this scale. One would expect short-term stability and good test-retest reliability over two to four weeks; but as the impact of allergy on the family can change in nature as children get older it would be useful to ascertain the sensitivity of the SPS-FA to longer term changes.

The study has some limitations which include the self-selected sample of participants who took part, who might be different in some way to parents who did not take part. We also had a sample that was predominantly mothers. This is typical of this type of research where it is very hard to recruit fathers, but it means that we do not know if this scale would demonstrate the same properties if fathers completed it; more research is needed with this group of people. We also had to rely on parents reporting the diagnosis of food allergy in their child. As this was an anonymous survey and the majority of children were prescribed adrenaline autoinjectors, there is no reason to suspect that parents did not report food allergy diagnoses accurately. However it would be useful to replicate the study with a sample recruited from allergy clinics to check that self-reporting of a diagnosis has not affected the results. Recruiting from a general population did allow for a large sample size of parents of children with a range of food allergy characteristics which is arguably more representative than recruiting from a newly diagnosed group.

In conclusion the SPS-FA is a reliable and valid scale for use in the UK and provides a holistic view of the impact of food allergy on the parent/child dyad. In conjunction with health-related quality of life measures, it can be used by health care practitioners to target care for patients. It will also enable the development and evaluation of psychological interventions for specific areas of need in the improvement of food allergy management and quality of life.

## Figures and Tables

**Table 1:** Characteristics of respondents (*n*%).

	Sample *n* = 434 *n*/%
Parents age (mean, s.d.)	42.21 (6.41)
Sex of parent completing survey	
Male	19 (4.4%)
Female	411 (94.7%)
Country of residence	
UK	410 (94.5%)
Other EU	12 (2.8%)
Non-EU	8 (1.8%)
Child age in years (mean, s.d.)	9.47 (4.7)
Child age range (years)	1–18
Sex of child with food allergy	
Male	282 (65%)
Female	148 (34.1%)
Number of children within family (mean, s.d.)	2.03 (1.12)
Number of children in family with a food allergy	
One	382 (88%)
Two	44 (10.1%)
Three	6 (1.4%)
Foods reported	
Peanut	335 (77.2%)
Tree nut	287 (66.1%)
Both peanut and tree nut	265 (54.1%)
Cow's milk	119 (27.4%)
Egg	162 (37.3%)
Soya	30 (6.9%)
Fruit	54 (12.4%)
Fish	32 (7.4%)
Sesame	43 (9.9%)
Wheat	16 (3.68%)
Shellfish	34 (7.8%)
Symptoms reported	
Vomiting	228 (52.5%)
Abdominal pain	155 (35.7%)
Rash, hives, urticaria	324 (74.7%)
Facial swelling	280 (64.5%)
Breathing difficulties	214 (49.3%)
Throat tightening	177 (40.8%)
Other allergies	
Latex	14 (3.2%)
Tree pollen	111 (25.6)
Grass pollen	121 (27.9%)
Asthma	310 (71.4%)
Eczema	366 (84.3%)
Hay-fever	240 (55.3%)
History of anaphylaxis	226 (52.1%)
Carries adrenaline autoinjector	411 (94.7%)
How allergy diagnosed	
Skin prick test	327 (75.3%)
Blood test	264 (60.8%)
Food challenge	66 (15.2%)
Hospitalisation due to an allergic reaction to food	282 (65%)

When % do not add up to 100 there are missing values; when % total more than 100 parents were able to select more than one answer.

**Table 2:** Relationships (Pearson's *r*) between the psychosocial impact of food allergy, quality of life, self-efficacy, mental health, FAIM scores, and demographic and food allergy characteristics.

	Psychosocial impact (SPS-FA)			
	Social impact	Crisis	QoL	SPS-FA total
Age of parent	−.19^*∗∗*^	−.06	−.17^*∗∗*^	−.18^*∗∗*^
Age of child	−.15^*∗∗*^	−.05	−.14^*∗∗*^	−.14^*∗∗*^
Number of allergies	.20^*∗∗*^	.22^*∗∗*^	.19^*∗∗*^	.25^*∗∗*^
Quality of life (FAQL-PB)	.59^*∗∗*^	.45^*∗∗*^	.66^*∗∗*^	.68^*∗∗*^
Food allergy self-efficacy (FASE-P)	−.46^*∗∗*^	−.34^*∗∗*^	−.41^*∗∗*^	−.49^*∗∗*^
Managing social activities	−.47^*∗∗*^	−.34^*∗∗*^	−.44^*∗∗*^	−.50^*∗∗*^
Precaution and prevention	−.39^*∗∗*^	−.26^*∗∗*^	−.32^*∗∗*^	−.39^*∗∗*^
Allergic treatment	−.15^*∗∗*^	−.05	−.15^*∗∗*^	−.14^*∗∗*^
Food allergen identification	−.10^*∗*^	−.08	−.14^*∗∗*^	−.12^*∗*^
Seeking information	−.28^*∗∗*^	−.22^*∗∗*^	−.22^*∗∗*^	−.29^*∗∗*^
General health questionnaire (GHQ12)	.32^*∗∗*^	.36^*∗∗*^	.53^*∗∗*^	.46^*∗∗*^
FAIM	.22^*∗∗*^	.19^*∗∗*^	.30^*∗∗*^	.29^*∗∗*^

^*∗*^ *p* < 0.05; ^*∗∗*^ *p* < 0.01.

QoL: quality of life; FAIM: food allergy independent measure.

**Table 3:** Differences in SPS-FA mean (standard deviation) scores in relation to food allergy characteristics.

	Psychosocial impact (SPS-FA)			
	Yes	No	*T* value	*p* value
History of anaphylaxis	3.45 (2.12)	2.80 (1.98)	2.94	0.013
Hospitalisation	3.46 (2.12)	2.68 (1.94)	3.76	<0.001
Presence of asthma	3.39 (2.14)	2.52 (1.68)	4.04	<0.001
Presence of eczema	3.35 (2.10)	2.38 (1.78)	3.16	0.002
Milk allergy	3.81 (2.08)	2.98 (2.06)	3.71	<0.001
Egg allergy	3.69 (2.02)	2.92 (2.10)	3.75	<0.001

**Table 4:** Hierarchical regression model showing significant predictors for the psychosocial impact of food allergy (SPS-FA scores) on the parent.

Predictors	Standardised *β*	
	Step 1	Step 2
Age of parent	−.09	−.05
Age of child	−.07	.09
Number of allergies	.20^*∗∗*^	.11^*∗*^
Anaphylaxis	−.05	−.04
Hospitalisation	−.14^*∗∗*^	−.05
Asthma	−.14^*∗∗*^	−.04
Eczema	−.06	−.04
Cow's milk allergy	.03	.09
Egg allergy	−.03	−.04
Food allergy QoL (FAQL-PB)		.52^*∗∗∗*^
Food allergy self-efficacy (FASE-P)		−.11^*∗*^
General health questionnaire (GHQ12)		.24^*∗∗∗*^
FAIM		−.02

*F* value	5.97	33.91
Adj *R* ^2^	.12	.56

^*∗*^ *p* < 0.05; ^*∗∗*^ *p* < 0.01; ^*∗∗∗*^ *p* < 0.001

QoL: quality of life; FAIM: food allergy independent measure.
